# Cannabis and Athletic Performance

**DOI:** 10.1007/s40279-021-01505-x

**Published:** 2021-09-13

**Authors:** Jamie F. Burr, Christian P. Cheung, Andreas M. Kasper, Scott H. Gillham, Graeme L. Close

**Affiliations:** 1 Human Health and Nutritional Sciences, University of Guelph, 50 Stone Road E, Guelph, ON N1G2W1 Canada; 2 Research Institute for Sport and Exercise Sciences, Liverpool John Moores University, Liverpool, UK

## Abstract

Cannabis is widely used for both recreational and medicinal purposes on a global scale. There is accumulating interest in the use of cannabis and its constituents for athletic recovery, and in some instances, performance. Amidst speculation of potential beneficial applications, the effects of cannabis and its two most abundant constituents, delta-9-tetrahydrocannabinol (THC) and cannabidiol (CBD), remain largely un-investigated. The purpose of this review is to critically evaluate the literature describing the effects of whole cannabis, THC, and CBD, on athletic performance and recovery. While investigations of whole cannabis and THC have generally shown either null or detrimental effects on exercise performance in strength and aerobic-type activities, studies of sufficient rigor and validity to conclusively declare ergogenic or ergolytic potential in athletes are lacking. The ability of cannabis and THC to perturb cardiovascular homeostasis warrants further investigation regarding mechanisms by which performance may be affected across different exercise modalities and energetic demands. In contrast to cannabis and THC, CBD has largely been scrutinized for its potential to aid in recovery. The beneficial effects of CBD on sleep quality, pain, and mild traumatic brain injury may be of particular interest to certain athletes. However, research in each of these respective areas has yet to be thoroughly investigated in athletic populations. Elucidating the effects of whole cannabis, THC, and CBD is pertinent for both researchers and practitioners given the widespread use of these products, and their potential to interact with athletes’ performance and recovery.

## Key Points

**Table:** 

Use of cannabis, THC, and CBD by athletes for the purposes of improving performance and recovery is increasingly reported across different sports and levels of competition
Appropriate empirical evidence regarding the effects of cannabis use on sport performance is lacking. Understanding the short- and long-term effects of cannabis and THC on human performance in athletes will require well-controlled, athlete-specific research, with applied performance outcomes
CBD may have some promise for aiding athletes with recovery by improving sleep quality, pain, and mild traumatic brain injury

## Introduction

The empirical case for or against cannabis use to aid athletic performance remains tenuous. Despite evidence of long-standing human consumption over the ages [1], scientific investigation into the effects of cannabis has been relatively limited, largely in part to challenges faced to investigate a drug that has a long global history of prohibition and tight regulatory control [2, 3]. Despite cannabis remaining an illicit drug in a majority of countries and holding a place on the World Anti-Doping Agency’s (WADA) prohibited substance list, accounts of its use amongst competitive and recreational athletes abound [4–7].

Today, the global use of cannabis and formulations made of its derivatives are progressively more widespread as many nations relax laws around both medical and recreational use. Cannabis has been noted as the second most consumed recreational substance, next to alcohol [8], and with such ubiquity and increasing belief of potential for health benefit, the enticement for uptake and use by athletes is not surprising. While commonly used as a recreational drug outside the context of sport, evidence suggests that athletes who use cannabis do so with the intent of enhancing performance (whether this is likely to occur or not), with factors such as sporting background, individual vs. team sport participation, competition level, sex, and demographic background further affecting predispositions to use [4–6]. A recent meta-analysis of 11 studies representing over 46,000 athletes of varying age and ability suggests that ~ 23% have used some form of cannabis in the past year [9].

Plants of the *Cannabis* genus contain over 100 genus-specific molecules [10], known as phytocannabinoids: the two most studied being delta-9-tetrahydrocannabinol (THC) and cannabidiol (CBD). THC is widely recognized for its psychotropic effects. The selective breeding and cultivation of new cannabis strains for both recreational and medicinal use has commonly focused on THC, resulting in alterations to cannabinoid ratios and increases in relative THC concentration over time [11]. It is now understood that THC exerts a variety of physiological effects via the endogenous cannabinoid, or endocannabinoid, system. More specifically, THC acts as a partial agonist to the putative endogenous cannabinoid receptors type 1 (CB1) and 2 (CB2), which are located in a wide range of central and peripheral tissues [12, 13]. CBD, on the other hand, is devoid of psychotropic effects [11], and while it appears to have limited physiological influence through CB1 or CB2, it is believed to act at a number of other receptor targets and may modulate the effects of THC [13–15]. Given the unique effects of THC and CBD to elicit differing physiological effects that could alter exercise performance or recovery, coupled with their differing status as legal or prohibited substances, there is a growing interest in scientific evidence for a variety of purported uses. Furthermore, exercise itself may have unique interactions with the endocannabinoid system which may modulate the effects of exogenous cannabinoids [16]. It is important to acknowledge that cannabis contains many other molecules which could theoretically have physiological effects, and consequently affect human performance; however, the effects of these less abundant cannabinoids and compounds are beyond the scope of this review.

Cannabis is most commonly consumed via inhalation of combusted plant material, colloquially referred to as smoking, which leads to rapid uptake and effects [17]. Cannabinoids may also be consumed by ingesting cannabinoid-containing food products, leading to delayed uptake (30–60 min post) with peak effects occurring between 1.5 and 3 h post-consumption [18]. It should, however, be acknowledged that the pharmacology of THC and CBD may vary significantly according to a variety of contextual factors, leading to a wide range of bioavailability and elimination rates, as reviewed elsewhere [19]. It is suggested that the effects of consumption, specifically the anxiolytic properties and muscle relaxing effects [4] are a highly sought-after effect for many athletes. Isolated CBD may also possess anxiolytic effects and is purported to have a variety of other beneficial effects such as improvements to sleep, exercise recovery, pain, anxiety, mood, and recovery from concussion [20]. These factors represent motivation for use amongst athletic populations, including professional athletes [20]. Despite the reported widespread use of whole cannabis, cannabinoid-based food products, and isolated CBD amongst athletes who have intentions of affecting athletic performance and/or recovery, there is no clear consensus about the general efficacy of use. At present, according to WADA, cannabis is in contravention of at least two of the three tenets of acceptable use in that it: has potential to enhance sport performance, represents a health risk to athletes, and violates the spirit of sport. Consequently, cannabis and all other cannabinoids (with the exception of CBD) are prohibited during the in-competition phase. Amidst the current evidence this remains a controversial topic in the anti-doping realm [21].

While opinions regarding the efficacy of cannabis (and its constituent products) to meaningfully affect sport performance remain split, cannabis demonstrates clear potential to perturb cardiovascular [22], respiratory [23], and cognitive function [24]. However, in an era of evidence-based decision making, a paucity of trials explicitly examining the effects of whole cannabis, THC and CBD on varied exercise performance and recovery specific outcomes leaves a significant vacuum in which decisions must be made. Notably, rulings about the suitability of use in the context of sport must simultaneously consider both the potential to alter performance and the potential for adverse health effects, which may include serious cardiovascular events, amongst other dangers, which are discussed in detail elsewhere [25–28]. Amongst these issues are the effects that THC may have on alterations in motor control or decision making [29] but in many ways these factors are limited to the psychological, as opposed to psycho-physiological, effects on performance. Thus, this review focuses on the existing evidence for the physiological effects of cannabis, THC, and CBD consumption for exercise performance and recovery, while highlighting requisite areas of future research to progress our empirical understanding in the context of sport performance. Given the notable differences in the psychological and physiological effects of THC and CBD, as well as the potential indications for use in the context of sport, CBD is discussed independently of whole cannabis and THC.

## Cannabis, THC, and Exercise Performance

The topic of cannabis use and the specific effects of THC on human exercise performance have been considered previously [9, 21, 27, 30–36]. Perplexingly, despite there being few new data generated over the past few decades, repeated interpretations of these data have led to vastly different conclusions, with reviews presuming: no benefit [30, 31], potential advantages [21] and predominantly an ergolytic effect [9, 32–34]. As such, we include a critical review of current work, and recognition of the knowledge gaps that must be filled to clarify the effect on human performance more specifically.

Compared to the empirical data on other performance related drugs and supplements, the evidence regarding cannabis and THC use is conspicuously lacking. While the volume of data is evidently sparse, it is also noteworthy that the most relevant literature was published 35–45 years ago, with little progress since. Further, there are important considerations of study design and collection methods that limit our ability to meaningfully extrapolate findings for understanding the potential effect of cannabis and THC use on sporting performance in the current day. Amongst these factors are: a substantial increase in the typical dose of THC over time (increased between sixfold and tenfold [11]), evolving methods of consumption and recognition that timing of intake/uptake may influence physiological responses, and an improvement in our ability to quantify performance as physical work output. At the time of writing, we have identified ten studies that consider the effects of cannabis on human performance. Four are cross-sectional studies that characterize the physical capacities of long-term cannabis users compared to non-using controls [37–40], and six involve the administration of cannabis or THC to participants prior to exercise [41–46]. Of these experimental studies, 50% were performed in persons with identified coronary artery disease or chronic obstructive pulmonary disease (COPD).

### Chronic Cannabis Users

Even at a superficial level, there is value in understanding whether individuals who habitually use cannabis differ in their ability to perform exercise from those who do not. Such investigations can shed light on the possibility of persistent performance effects of cannabis with long-term use. However, while such research designs eliminate the need for logistically challenging studies that require the longitudinal administration of dosed cannabis, their cross-sectional nature precludes the control of potential bias and cause-and-effect cannot be concluded. Of the existing data comparing cannabis users to non-users, there are no reported differences in aerobic fitness (*V*O_2max_), blood pressure, muscular strength and endurance measures, work capacity, and perceived exertion [37–40]. In physically active cannabis users, there are no differences in anaerobic power, or markers of stress and inflammation [37, 39]. Notably, in all studies, participants had been asked to abstain from cannabis consumption for hours to days prior to testing in an attempt to avoid transient physiological effects from recent use, but this might not represent a normal functioning state for heavy users and may be confounded by potential interactions with withdrawal effects [47], which should also be considered in studies where cannabis is administered. The typical concern about the potential for bias (e.g., self-selection to participate) in cross-sectional studies must still be considered. Taken together, there is presently little evidence to suggest chronic cannabis use performed in isolation from training or competition exerts a great effect on any measure of physical performance in recreationally active participants.

### Diseased

Exercise is a commonly used tool for the identification and elicitation of signs and symptoms of underlying cardiovascular disease (CVD). While studies examining the combined cardiovascular stress of exercise with cannabis use in persons with identified CVD offer select insight into the human ability to perform aerobic work, their widespread acceptance as concrete evidence of the effects of cannabis or THC on general or high-level human performance is likely misplaced. It is worth highlighting that these studies were never designed to specifically address these questions, and this fact appears to be commonly overlooked in the context of interpretation and application for sport.

The first study of cannabis and exercise was performed in patients (*n* = 10) with significant coronary artery disease (> 75% narrowing of coronary artery), with the onset of angina as a major endpoint [42]. Comparing exercise capacity after smoking cannabis or a cigarette placebo, both groups demonstrate a decrease in time to exhaustion. This effect was greater with cannabis use (48% vs 8.6%), possibly because of an increase in myocardial oxygen demand (rate-pressure product), a mechanism discussed in Sect. 3. It is worth noting that, according to the loading protocol described in the methods, even during the control condition participants were capable of only a 25 W power output and ≤ 25% of the test was performed at an increased workload of 50 W. For comparison, the modern professional male cyclist can sustain approximately 500 W for a similar duration of 120 s amidst hours of riding on consecutive days [48], highlighting the absurdity of using one population to predict effects in the other. The use of cannabis was also a novel stimulus amongst these participants. This work was confirmed by the same group using similarly diseased patients, and an equally low exercise stimulus and THC dosage (< 15 mg) the following year [45]. It is worth highlighting that these two studies commonly represent > 50% of the “performance” evidence cited in existing reviews to suggest an ergolytic effect of cannabis on performance. More recently, to explore the effects on breathlessness and exercise capacity, COPD patients consumed whole cannabis (vaporized not smoked, 6.4 mg THC) prior to cardiopulmonary exercise testing [46]. Cannabis was reported to have no impact on any cardiorespiratory responses, nor exercise time, and it is worth noting that exercise lasted < 5 min. Importantly, this group of patients had advanced COPD and fitness that was approximately 10% of what would be expected for a healthy (non-athletic) control.

### Healthy

Steadward and Singh published the first investigation—and to date one of only three studies—in healthy participants (*n* = 20), exploring the effects of cannabis on cardiorespiratory responses to exercise, physical work capacity, and strength [41]. After smoking a moderate dose of THC (18.2 mg), participants showed no apparent effect on handgrip strength, but submaximal work capacity on a cycle ergometer decreased. Importantly, this decrease was concomitant with and inseparable from an increase in submaximal heart rate (HR) in the cannabis group. In papers reporting this research as evidence of an ergolytic effect of cannabis, it is commonly ignored that the test of physical performance, known as the PWC_170_—a commonly used test from this time, is a submaximal test for which performance is estimated based on the work output at a given HR (i.e., physical work capacity at a heart rate of 170 bpm). While the linear relationships between HR and either *V*O_2_ or power output make this a useful measure of capacity under normal circumstances [49, 50], this relationship can no longer be trusted as accurate after the administration of a drug that specifically alters the variable that is being controlled for. It should be surprising to no one that when submaximal HR is artificially inflated—and power is measured at a clamped HR—that the work output will necessarily be lower. Notably, there are examples of other definitively ergogenic substances, such as ephedrine and caffeine, that increase submaximal HR and improve endurance performance [51, 52]. Thus, it is inadvisable to conflate the tachycardic effects of cannabis consumption with an ergolytic effect. Avakian et al. [43] similarly followed-up with a submaximal exercise study (40–50% *V*O_2max_) with individuals (*n* = 6), habituated to cannabis use. While no effects of cannabis use were evident on blood pressure, ventilation (V̇E) or *V̇*O_2_, a sustained tachycardic HR response was reported. However, no true measures of maximal exercise performance were recorded and the extrapolation of a submaximal effect on HR at 50% of capacity to exercise performance in a competitive situation requires large, unsupported assumptions of equivalency. Despite the results of this work often being referenced regarding exercise performance, the authors themselves conclude that “…the significance of their observation is not established”. Interestingly, all subjects were able to identify the placebo from the low dose (7.5 mg) THC condition during exercise, leaving the possibility that psychological factors could modify exercise behaviors, but this has yet to be an empirically tested outcome, nor has blinding been effectively performed. This is an important consideration for future research examining the psycho-physiological effects of cannabis in a more targeted performance setting.

The final, and most specific performance work to date, is the only study to examine healthy participants exercising to maximal capacity. In this work, Renaud and Cormier [44] had participants (*n* = 12) perform progressively more challenging workloads (16.4 W each min) until “leg failure” both under control conditions and after having consumed cannabis (1.7% THC) dosed at 7 mg of dried cannabis per kg body weight. At maximal exercise, no differences were found in HR, *V̇*E, *V̇*O_2_ or volume of exhaled carbon dioxide (V̇CO_2_); suggesting that despite the submaximal tachycardic response, physiologic responses at maximal workloads were not different after cannabis consumption [44]. Examination of submaximal through maximal work clearly shows a diminishing difference between placebo and cannabis groups as exercise intensity is increased. At workloads greater than 80% of maximal effort no differences existed, calling into question the implications of previous submaximal work for modelling effects of performance, especially as *V̇*O_2max_ is not affected [44]. The most lauded finding from the work of Renaud and Cormier [44] was the fact that there was a significant difference in exercise duration, with cannabis exposure decreasing time to exhaustion. While the data do indeed support this finding, examination of the exercise testing protocol demonstrates that this difference (16.1 ± 4 vs 15.1 ± 3 min) represents an average difference of a single one-minute stage, and 100 kpm/min, or about 16 W. It is unclear if this was truly a scaled linear variable (time), or if it was an ordinal variable—such that participants were encouraged to finish each stage with only finished stages being counted. In either case, the implications of such a small magnitude change on this type of staged test are questionable. As this is truly the only investigation of exhaustive performance in healthy participants, the methodological ambiguity and debatable practical validity of the findings indicate that further work is warranted.

### Knowledge Gaps and Recommendations

There is a paucity of valid exercise studies designed to specifically investigate the effects of cannabis and THC on human exercise capacity and performance. It is noted that physiological capacity and performance are interrelated, but not equivalent. Factors such as an athlete’s perception (e.g., time, pain, appropriate pacing strategies) and motivation ultimately affect performance and could also be altered by cannabis or THC, but have not been investigated. Healthy subjects who have varied levels of fitness and habituation to cannabis need to be studied, with further consideration of the methods of cannabis intake and the pharmacokinetics that dictate the time of peak effects. Dose–response curves should be developed, with varied exercise modes (e.g., cycling, running, strength assessments), intensities (i.e., submaximal, maximal, sprint-power based), durations (e.g., up to and including ultra-endurance events) and models of performance (time to exhaustion, time trial, power output). Improved technologies, including electronically braked ergometers and breath-by-breath indirect calorimetry, now allow research to move beyond the incremental-stage graded exercise test. These contemporary technologies and best practices should be employed to increase the sensitivity and validity for tests of physical capacity and performance.

## Cannabis and Systems-Level Cardiorespiratory Physiology

While the bona fide effects of cannabis on athletic performance are limited by an incomplete evidence-base with low ecological validity for athletes, the physiological actions of cannabis and THC offer important insight into perturbations of cardiorespiratory homeostasis through which cannabis may interact with performance. Studies from these areas have generally been considered separately (for reviews of cannabis and performance see [9, 21, 27, 30–36]; for reviews of cardiovascular effects of cannabis see [22, 25]) with no comprehensive integration. As exercise clearly requires a coordinated and integrated response from multiple physiological systems, this is a notable shortcoming.

Studies administering cannabis and isolated THC to healthy individuals have revealed a wide range of cardiovascular effects, including: changes in heart rate [41–45, 53–85], cardiac function [55, 59, 64, 75, 76, 79, 83], blood pressure [41, 42, 53, 57, 59–61, 63–65, 75–78, 83], orthostatic hypotension [53, 60, 80], ventilatory sensitivity to carbon dioxide [61], and limb blood flow [53, 78, 85].

Transient sinus tachycardia is a commonly reported dose-dependent effect of cannabis and THC consumption [55, 68, 70, 72, 82, 83]. As noted for the performance literature, this effect persists during submaximal exercise, resulting in a greater rate-pressure product at a given exercise intensity, indicative of increased myocardial oxygen demand [86]. When consumed via smoking, this elevated myocardial oxygen demand associated with cannabis consumption could be exacerbated by reduced oxygen supply consequent to the inhaled carbon monoxide present in cannabis smoke [87]. The disruption of the myocardial oxygen supply/demand relationship likely explains why myocardial ischemia is precipitated by cannabis smoking in coronary heart disease patients [42, 45]. It is worth noting this phenomenon may not be unique to cannabis smoking, as placebo and traditional cigarette smoking in these same studies induced an attenuated, but still significant, reduction in time to angina compared to cannabis, likely due to the fact that all smoking involves the inhalation of carbon monoxide and hydrocarbons but does not produce the cannabis specific tachycardic effect.

The acute effects of cannabis and THC consumption on blood pressure are more variable, with potential implications for perfusion during exercise. Investigators have reported increases in systolic and diastolic pressure [41, 42, 53, 59, 61, 64, 65, 76–78, 83], reductions in blood pressure [60, 63], or no changes in blood pressure [43, 58, 59, 62, 63, 73–75, 81, 88–90] following cannabis or THC consumption. Unlike HR, it appears that pressure responses during exercise are not affected following a single instance of cannabis consumption [43]. However, if THC is persistently administered in high doses (up to 210 mg/day for multiple weeks) blood pressure responses to exercise are altered; with an attenuation of the rise in systolic blood pressure, and an exaggeration of the reduction in diastolic blood pressure [77]. Thus, the timing and quantity of THC dose may also influence the pressor response to exercise. The reported increase in limb blood flow following cannabis consumption [53, 78, 85] could also partially explain the varied effects on pressure, but limb blood flow following cannabis and THC consumption has only been examined at rest. During exercise performance, cannabis induced increases in flow could be relevant given limited evidence that suggests that muscle blood flow may, to some degree, limit maximal exercise capacity [91]. Further studies are needed to reach conclusions about how these hemodynamic effects of cannabis and THC interact with performance outcomes.

Echocardiographic studies have generated equivocal findings with respect to cardiac function, reporting both reduced systolic function [59] and increased left ventricular tissue velocity [64, 76] following cannabis smoking. Whether the myocardial effects of cannabis and THC impact exercise performance remains unclear, owing largely to uncertainty over whether the effects observed at rest persist during exercise—and if so, the extent of functional consequences. Employing modern tests of cardiac function and imaging techniques, such as stress echocardiography [92] or magnetic resonance imaging [93], is required to fill this knowledge gap, as imaging and analysis capabilities have evolved substantially since the publication of the aforementioned seminal works. The importance of cardiac mechanics (such as left ventricular strain, torsion and twist) for understanding cardiac function is increasingly recognized [94, 95]; yet to date only two studies have examined these variables in chronic cannabis users [96, 97], and no studies have examined the acute effects of cannabis or THC.

A large body of evidence from *in-vitro* and animal models indicates that the mechanisms through which cannabis and THC elicit cardiovascular effects are likely vast and many could be relevant to exercise performance (for review, see [25]). In humans the cardiovascular effects of THC and cannabis appear to be largely mediated through autonomic nervous mechanisms and the endocannabinoid system. Treatment with beta-adrenergic blockade prior to THC or cannabis administration markedly blunts tachycardic [65, 75, 84, 85] and pressor [65, 75] responses, increases in limb blood flow [78, 85], and alterations to systolic time intervals [75]. Thus, it appears that the cardiovascular effects of THC and cannabis are mediated at least partially through the sympathetic nervous system. Beta-adrenergic blockade in combination with anti-cholinergic agents augments the effects of THC and cannabis on HR and blood pressure [75, 77, 78, 85]. The endogenous cannabinoid system also appears able to facilitate certain cardiovascular effects of THC, suggesting a degree of redundancy. Following the discovery of the endocannabinoid system and identification of CB1 in cardiovascular tissues [98, 99], investigations of human participants revealed that inhibition of CB1 also ablates the tachycardic effects of cannabis [69, 70, 72–74, 89, 100]. Despite these putative mechanisms through which cannabis and THC exert cardiovascular effects, cannabis contains hundreds of chemical compounds, including over 100 phytocannabinoids [10]. Thus, it should be accepted that the physiological effects of cannabis cannot be solely attributed to THC until further investigations examine the many constituents of the plant.

Given the inseparable links of the cardiovascular and respiratory systems to support aerobic exercise performance, the effects of cannabis and THC consumption on respiratory function must be considered. Epidemiological analyses of the respiratory effects of chronic cannabis use have failed to show a clear linear relationship between cannabis smoking and reduced pulmonary function [101], and generally only demonstrate reduced function with very heavy cannabis use [102]. Thus, it may be that exercise capacity is, similarly, only impacted negatively with heavy cannabis use. Cross-sectional data comparing *V*O_2max_, work capacity, pulmonary function, and strength and endurance outcomes have consistently demonstrated no differences between young cannabis users and non-users [37–40]; however, no comparison has been made between non-users and longtime heavy users.

The acute and transient effects of cannabis and THC on respiratory function during exercise have received little attention. Of the two studies performed in healthy individuals, one revealed no differences in respiratory function [41], and the other demonstrated an increased capacity to expire forcefully (FEV_1_) after exercise [44]. Alterations in flow are most likely related to the reported bronchodilator effects of THC [103]. Theoretically, the ability of THC to induce bronchodilation in healthy and asthmatic participants [104] provides a potential mechanism through which cannabis could influence performance, as bronchodilating substances have previously been used by athletes for purported ergogenic effects [105]. However, the ergogenic potential of this bronchodilator effect remains to be confirmed, and it is notable that COPD patients experienced no improvement in exercise capacity or respiratory function following inhalation of vaporized cannabis [46]. It must also be considered that a number of bronchodilating substances are currently permitted for use in sport by WADA [106], and the evidence supporting the ergogenic effects of bronchodilating substances is tenuous [107]. Another intriguing observation is that THC appears to alter ventilatory sensitivity to carbon dioxide [61]. At present, it is not clear whether this effect occurs, or is consequential, during exercise.

There currently exists a substantial body of evidence demonstrating that consumption of cannabis leads to numerous systemic cardiorespiratory effects at rest, largely due to the actions of THC. Despite increasing use of recreational and medicinal cannabis, it remains unclear which effects persist during dynamic exercise, and how they might impact performance. Understanding these effects is not only necessary to determine how cannabis impacts performance across athletic disciplines, but also to inform decisions regarding the safety and regulation of cannabis use in both athletic and non-athletic populations.

### Knowledge Gaps and Recommendations

The effects of cannabis and THC on rudimentary cardiorespiratory physiology in resting humans are well characterized; however, both cannabis potency and investigative research capabilities have increased dramatically since many of these studies were initially performed, without concomitant study. The independent effects of inhaling combusted plant material versus the effects of cannabis and THC are incompletely understood, both at rest and in an exercise context. For future work to successfully characterize the effects of cannabis and THC on performance, underlying physiological effects must be rigorously investigated before, during, and after exercise.

## CBD and the Elite Athlete

While whole cannabis is commonly used across the globe for both recreational and medicinal purposes [9] in many countries it remains an illicit drug [108], and from an athletic perspective it is currently prohibited (in competition) by WADA. However, one of the major phytocannabinoids within cannabis, CBD, was specifically removed from the WADA prohibited list in 2018 and as a consequence its interest in sport has grown exponentially. Interest in CBD is also largely driven by the fact that the l-isomer of CBD originating from cannabis plants does not have psychotropic properties, although some synthetic analogs might [109]. The use of CBD in sport has recently been reviewed extensively [110]. The individual phytocannabinoid derivatives from the cannabis plant face discrepant restrictions by some sporting governing bodies and WADA [106]. Of all the phytocannabinoid derivatives, the only constituent absolutely legal from a WADA perspective is CBD. All other phytocannabinoid derivatives are prohibited per se except THC which is considered a threshold substance, meaning that only concentrations > 150 ng/ml in urine result in an anti-doping rule violation (ADRV) [111]. It is also important to stress that the legislative regulation of CBD itself is somewhat complex and it is, therefore, vital that athletes are aware of the specific country, and in the case of the US, state specific legislation before considering the potential for CBD use in sport. Despite this lack of clarity surrounding the precise legality of CBD commerce and consumption, supplementation in athletic populations has grown due to its purported effects on athletic performance and recovery [9, 20].

### Sleep and Anxiety

Appropriate sleep is widely accepted as an integral component of the recovery process in athletes (for review see [112]). Professional athletes have previously reported sub-optimal sleep quantity [113] and quality [114]. Indeed, disturbances in sleep can be a consequence of several mechanisms including pre-game supplementation [115], the time of competition [116], implications of long-haul travel [117], and anxiety associated with competition [118–121]. It is, therefore, understandable that athletes supplement products such as CBD, with the aim to improve sleep efficiency and provide anxiolytic properties [122], despite associated evidence being limited to clinical research as opposed to within elite athlete cohorts.

Any potential positive effects of CBD on sleep are primarily limited to diseased populations, such as sufferers of Parkinson’s disease [123] and post-traumatic stress disorders [122], with randomized controlled trials in human participants somewhat limited. However, Carlini & Cunha reported that CBD supplementation (160 mg) significantly increased sleep duration in individuals reporting difficulties in both sleep onset and quality [124]; however, conclusions were limited to perceived/subjective measures as opposed to objective polysomnographic data. More recent research from Linares and colleagues showed no significant effects of CBD (300 mg) on either subjective sleep quality or objective polysomnography measures, though it is important to note that although the latter utilized a higher dose of CBD, participants were healthy and not experiencing any reported sleep disturbance [125]. As such, although CBD shows promise in sleep quantity and quality, well-designed randomized controlled studies in athletic populations are required to determine the exact, if any, situation in which CBD may provide this sleep (and thus recovery) enhancing effect.

### Pain, Inflammation, and Muscle Function

Exercise induced muscle damage (EIMD) is a well-established phenomenon following athletic activity (for review see [126]). Throughout congested competition schedules and particularly damaging exercise bouts [127], pain and recovery management is often modulated via non-steroidal anti-inflammatory drugs (NSAIDs) and, in some cases, opiates [128]. However, in addition to the adaptation blunting effect of NSAIDs [126], chronic consumption has the potential to induce several adverse health effects [129]. It is, therefore, unsurprising that in a recent study of elite rugby players, 26% of players had previously experimented, or were currently using CBD supplements, which have been shown to have mild-moderate adverse effect profiles in humans [130, 131]. That said, as a result of CBD’s metabolism by the CYP3A4 and CYP219 enzymes it can potentially increase drug-drug interactions with other compounds metabolized by the same enzymes, subsequently increasing potential adverse effects profiles [132]. In this study, ~ 80% of rugby players cited pain management as their primary motive for experimenting with CBD [20]. This high prevalence of use was in spite of the current lack of an evidence-base for the efficacy of supplementation, and risks of potential ADRVs [20, 133]. Interestingly, despite the high number of rugby players taking CBD for pain modulation, only 14% of rugby players reported any beneficial effect [20]. These findings may be as a result of the disparity in the reported doses consumed by athletes [20], especially as higher doses may be required to offer anti-inflammatory effect in humans. Low vs. high doses of CBD (10 vs. 500 mg/day) have shown differing pain alleviating results (non-significant vs. significant) in patients experiencing high levels of gastrointestinal inflammation [134, 135], with higher dosages, although significantly relieving pain, also resulting in issues within the gastrointestinal tract or central nervous system. It is worth noting that the population of this study already had gastrointestinal issues, which may, in part, explain these increased adverse effects [135].

Despite the widespread inclusion of strength training amongst high-level athletes, studies investigating the effects of CBD supplementation on resistance exercise are extremely limited. To date, only two preliminary studies are available with varying research designs, and equivocal findings. For example, 150 mg day of oral CBD (2 × 75 mg doses given immediately post, 24 and 48 h following a muscle-damaging protocol) had no beneficial effects on either muscle function or perceived soreness in untrained males (*n* = 13) [136]. The only other available data on CBD and muscle soreness in an athletic context are limited to a single abstract from a conference communication [137]. This study assessed muscle damage (via creatine kinase [CK]) following a single bout of resistance exercise and suggested that CBD supplementation (60 mg/day) attenuated the acute increases in CK. However, alongside the proposed reduction in muscle damage, there were also reductions in strength within 24 h of supplementation. It is important to consider that neither of these studies assessed blood or urine cannabinoid concentrations and the equivocal data could be related to the efficacy of the supplementation protocols with major differences in the actual dose of CBD, number of days supplemented, and route of administration. Collectively, the evidence to date on the effects of CBD on muscle function following damaging exercise could be, at best, described as ‘in its infancy’ and, therefore, it is not possible to reach any form of conclusion as to the efficacy of CBD for muscle recovery. Research is now required, including pharmacokinetic data, measures of blood cannabinoids, and dose–response data to fully explore if CBD is able to attenuate muscle damage and/or enhance recovery following exercise.

### Neuroprotection and Traumatic Brain Injury

Concussion is a type of mild traumatic brain injury (mTBI) [138] which may occur following a rapid deceleration or rotational force applied to the brain [139]. These biomechanical mechanisms of injury are a particular concern in collision and combat sports such as rugby union [140], rugby league [141] American football [142, 143], as well as boxing and mixed-martial arts [144]. Several acute side-effects may be experienced during concussion including headaches, cognitive impairments, sleep disruption, and behavioral changes [145]. Moreover, long-term effects of concussion can include behavioral changes leading to aggressive episodes, anxiety, and depression [146]. Despite the exact mechanisms by which this may be achieved being unconfirmed, CBD has been proposed to offer a protective benefit in athletes who are “at risk” of mTBI in sport [147]. Suggested mechanisms include the anti-inflammatory nature of CBD [148, 149], anandamide uptake and enzymatic hydrolysis [150], and/or a decrease in adenosine reuptake [151]. To date, a single murine study has investigated the effects of CBD on mTBI [147]. In this study the authors concluded that chronic CBD administration (equating to ~ 51 mg/day when converted to a human equivalent dose [152]) reduces dysfunctions relating to the anxious, aggressive and depressive behaviors often exhibited following mTBI. Given the severe consequences of mTBI to health, coupled with the proposed neuroprotective potential of CBD, it is imperative that additional investigation in this area be completed in humans to understand the mechanisms by which CBD may offer a neuroprotective benefit to athletes who are at risk of mTBI.

### Knowledge Gaps and Recommendations

As a consequence of the complicated legislative status of CBD, research *in-vivo* is less common than for other ergogenic supplements regularly consumed by athletes. Whilst many CBD products available for purchase without prescription claim to have negligible, or even 0% THC, these claims are sometimes unfounded with a recent study suggesting that only 3 of a selected 25 CBD products were within ± 20% of claims made on their respective containers [153]. Moreover, many CBD products that are THC free still contain other cannabinoids, which are prohibited by WADA, and detection may result in ADRVs. Indeed, as THC can be stored in fat tissue [154], blood and urine metabolites may peak following specific periods of lipolysis inducing exercise [154] or fasting [155]. When considering CBD products athletes should also ensure that the L-isomer is the molecule contained, and be aware of the potential presence of other CBD analogs, which could possess psychotropic properties that may not be desired [109]. It is also important to consider that where CBD has been suggested as the root cause of significant findings, there may in fact be an ‘entourage effect’ as other cannabinoids may be present [156]. From a safety perspective, despite being reported to have a reasonably low adverse effects profile, there appear to be significant drug metabolism interactions, as CBD is metabolized by CYP450 isoforms 2C19 and 3A4 [157, 158]. Approximately 60% of clinically prescribed medications are metabolized by CYP3A4 and as a consequence there are suggestions that CBD can increase the adverse effects profile of standard medications such as clobazam used to treat epilepsy [130]. Subsequently, future research should investigate the efficacy of CBD in its therapeutic role in pain and recovery management, sports-related anxiolytic and sleep promoting effects, and examine drug interactions and side effect profiles of CBD supplementation. It is essential that studies begin to further investigate the mechanistic properties of CBD (and any ‘entourage effect’), as well as explore translatable mechanistic findings in-vivo.

## Conclusions

Cannabis and individual cannabinoids have a clear capacity to affect certain facets of human physiology; however, the applicability of such physiological perturbations to affect improvements in the health, performance, or recovery of athletes remains incomplete owing to large knowledge gaps and low-quality existing evidence stemming from substantial barriers to conducting high quality cannabis research [2, 3]. Herein, we have provided an overview of the existing evidence and areas for future research. Unlike CBD, cannabis and THC are prohibited by WADA in-competition, and while the cardiorespiratory effects at rest of cannabis and THC are well described, both the short- and long-term effects on the human capacity for exercise require well-controlled, athlete-specific research, with applied performance outcomes. Additional work will be required to understand the dose–response of these effects, accounting for methods of consumption, timing around exercise and cannabinoid concentrations. Such data are essential for weighing the evidence for or against prohibition both in and out of competition. The use of CBD by athletes is likely more relevant to recovery during training and while in competition. CBD may have some promise for improving athlete pain and recovery through a number of potential mechanisms, although evidence to support this to date is extremely limited. Moreover, the use of CBD requires prudent attention to local regulations and contamination with prohibited cannabinoids could trigger doping violations.
